# Area-Dependent Resistive Switching and Interfacial Dynamics in GCMO-Based Memristors

**DOI:** 10.1021/acsaelm.5c00403

**Published:** 2025-04-23

**Authors:** Anni Antola, Johanna Laaksonen, Hannu Huhtinen, Ilari Angervo, Sari Granroth, Alejandro Schulman, Pekka Laukkanen, Petriina Paturi

**Affiliations:** † Wihuri Physical Laboratory, Department of Physics and Astronomy, University of Turku, FI-20014 Turku, Finland; ‡ Materials Research Laboratory, Department of Physics and Astronomy, University of Turku, FI-20014 Turku, Finland; § Facultad de Ciencias, University of Salamanca, 37008 Salamanca, Spain

**Keywords:** GCMO, memristor, area-dependence, X-ray photoelectron spectroscopy, XPS

## Abstract

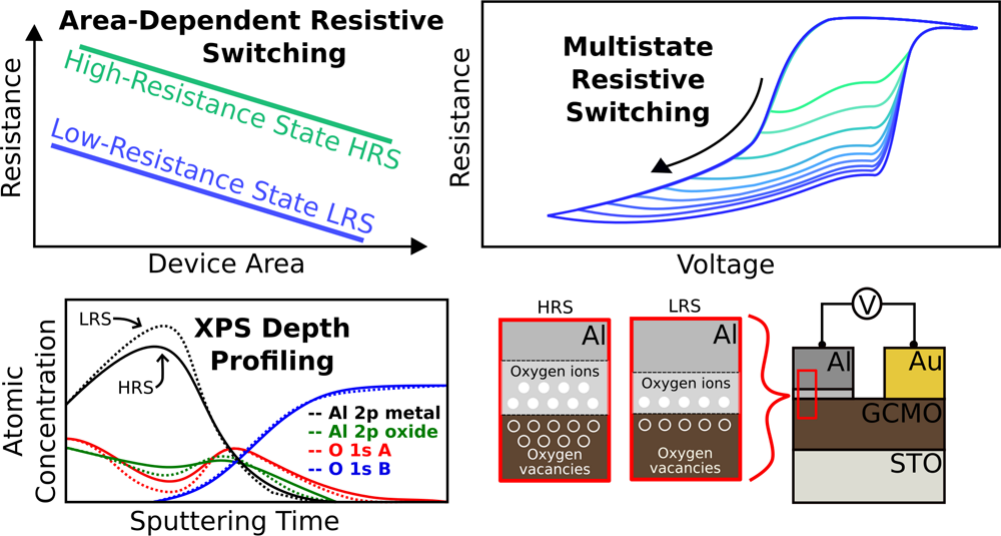

This study explores the area-dependent resistive switching (RS) characteristics of Gd_0.2_Ca_0.8_MnO_3_ (GCMO)-based memristors with aluminum (Al) and gold (Au) electrodes, emphasizing their potential for neuromorphic computing applications. Using a combination of electrical measurements and X-ray photoelectron spectroscopy (XPS), we demonstrate that the high-resistance (HRS) and low-resistance (LRS) states exhibit predictable scaling with device area, with HRS resistances ranging from 10^7^ to 10^8^ Ω and LRS from 10^5^ to 10^7^ Ω, supporting the hypothesis of interface-type RS. XPS depth profiling revealed notable differences in AlO_*x*_ interfacial layer composition between HRS and LRS, with a higher oxide content and a widened interfacial region in HRS. Additionally, the multistate RS capability of up to ten distinct levels was achieved by modulating applied voltages, highlighting GCMO’s suitability as a material for synaptic weight storage in artificial neural networks. Our findings underscore GCMO’s promise for energy-efficient, scalable memristor-based systems.

## 1 Introduction

Resistive switching (RS) in oxide-based memristors has emerged as a promising approach for neuromorphic computing applications, utilizing nonvolatile memory properties, analog control, and scalability.^1−5^ These features make oxide-based devices among all RS devices (also known as ReRAM, RRAM, or resistive random-access memories), particularly well-suited for emulating synaptic functions in artificial neural networks (ANNs).^1,2,5^ These materials are often based on the valence change mechanism (VCM), where an oxygen-rich, insulating material is transformed to be more conductive through modifying the oxygen ion concentration. Such transformation can be seen, for example, between perovskite structured insulating SrFeO_3_ and the counterpart SrFeO_2.5_,^6^ where the movement of oxygen ions corresponds with the interface-type RS resulting in the analog nature. VCM is also responsible for the filamentary-type RS behavior, as observed in widely studied HfO_*x*_-based memristors.^7−9^ Hafnium oxide is considered a benchmark material for VCM memristive devices due to its robust resistive switching characteristics, high endurance, and compatibility with existing semiconductor technologies. Recent studies have also highlighted the importance of interface engineering and material optimization for enhancing analog synaptic functionality.

Across the range of different oxide-based switching layer materials, perovskite manganite oxides (or manganites) with the general formula R_1–*x*_A_*x*_MnO_3_ (R = rare earth, A = alkali or alkaline earth metal), known for their mobile oxygen vacancies, allow for the formation of tunable resistance states through electric field-driven vacancy migration.^2,5,10^ Compounds such as Pr_1–*x*_Ca_*x*_MnO_3_ (PCMO), La_1–*x*_Ca_*x*_MnO_3_ (LCMO), and La_1–*x*_Sr_*x*_MnO_3_ (LSMO) demonstrate compliance-free and forming-free valence change-based, interfacial RS behavior,^11−17^ overcoming challenges such as the stochasticity and yield issues associated with high forming voltages typically seen in filamentary RS systems.^2,18^ Furthermore, the scalability of these materials’ resistances with device area—an indicator of interface-type RS^2,19^—supports their integration into high-density crossbar arrays, providing a structural foundation for large-scale synaptic networks.^20,21^

Among perovskite manganite materials, Gd_1–*x*_Ca_*x*_MnO_3_ (*x* = 0.8, GCMO), has shown compelling RS behavior for artificial synapse applications due to its stable, interface-driven RS characteristics.^22−26^ GCMO-based memristors have reached retention up to 8 h and endurance up to 10^5^ switching cycles.^22,24^ The specific doping level of *x* = 0.8 in GCMO has achieved a favorable balance between lower bulk resistivity and higher RS ratios, aided by a low Poole-Frenkel trap energy of 0.3 eV.^22,27^ The unique characteristics, such as the unusually high optimal cation doping and elevated interface resistance in a memristor, set GCMO-based devices apart from other studied manganites, indicating promising avenues for future research and potential energy-efficient device applications exclusively offered by GCMO. This study aims to clarify the origin of the RS mechanism in GCMO and further refine its suitability for neuromorphic computing. We first examine the dependence of GCMO’s high-resistance state (HRS) and low-resistance state (LRS) on device area to support the hypothesis that the RS observed in GCMO is interface-type, wherein resistance scales predictably with the area. Verifying this property would highlight GCMO’s suitability for densely packed crossbar architectures, a key configuration for neuromorphic networks.

In GCMO-based memristors, the RS mechanism is believed to be dominated by oxygen vacancy migration at the interface between the GCMO layer and an active electrode, for example, aluminum.^22^ Materials such as PCMO have been verified to follow the oxygen diffusion model with oxygen vacancy buildup near the active interface under an electric field,^12,28^ or correspondingly oxidation of the active electrode.^29^ In the Al/GCMO memristor, this interface-driven switching process is assumed to rely on the AlO_*x*_ (or Al_2_O_3_) interfacial layer forming at the active interface, a high-resistance barrier that can be modulated with chosen voltage pulsing schemes. This interfacial layer and potential alterations in the GCMO material properties near the interface are central to our analysis. To investigate these dynamics, we employ X-ray photoelectron spectroscopy (XPS) to assess differences in oxygen vacancy concentration and chemical composition between HRS and LRS states, thus clarifying the oxygen ion and vacancy redistribution mechanisms underlying RS.

Additionally, we explore the possibility of achieving multistate RS in GCMO devices by modulating applied voltages, particularly manipulated negative (SET) voltage limits. Such multistate RS, also known as multilevel or multibit RS, is of significant interest for neuromorphic computing, as it enables GCMO memristors to act as analog synapses, adjusting conductance states for continuous synaptic weight modification in NNs. By examining these characteristics, this study aims to establish GCMO as an efficient, scalable, and stable material for next-generation neuromorphic systems.

## 2 Experimental Section

A 100 nm thick GCMO film was deposited on a 10 mm × 10 mm SrTiO_3_ (100) (Crystal GmbH) single-crystal substrate using pulsed laser deposition (PLD), as described in our previous works.^30,31^ The GCMO film was patterned into varied-width crosspoints using maskless, laser-writer-assisted (Dilase 250, Kloé) photolithography and wet chemical etching. The chosen widths were 50, 100, 200, and 300 μm.

**Figure 1:**
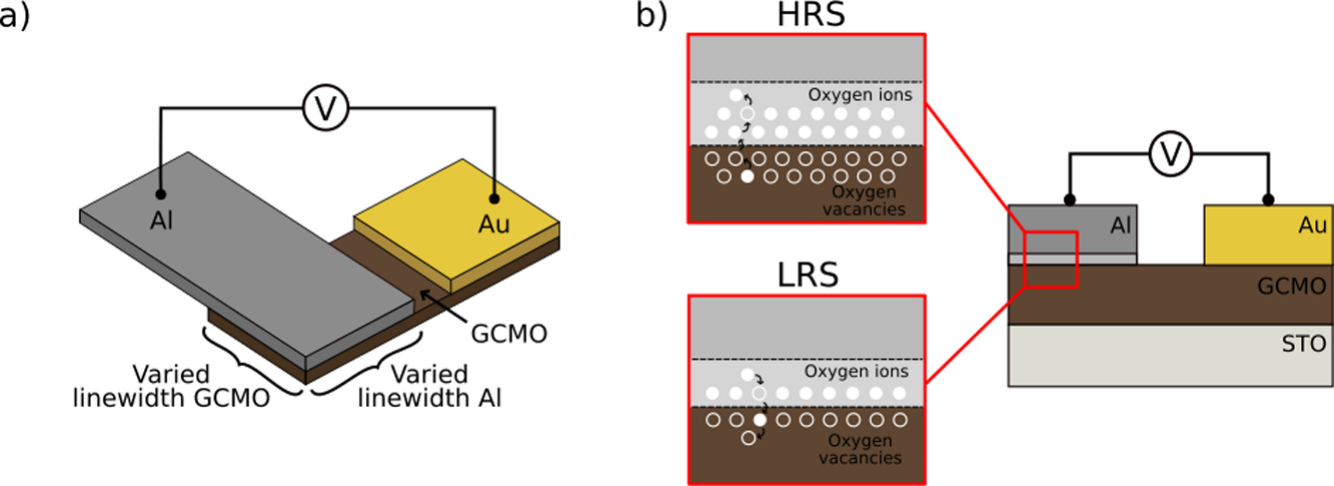
Schematic pictures of (a) Al/GCMO/Au memristor device configuration and (b) cross section of the device depicting oxygen ion/vacancy movement at the Al/GCMO interface. The thicknesses or sizes of the layers are not in scale.

For the Ohmic electrical contacts for the area-dependent devices, 50 nm thick Au pads were deposited at the ends of the GCMO patterns with electron beam evaporation (EBPVD, Elettrorava) and a lift-off process utilizing photolithography. To ensure optimal surface quality and cleanliness, a 30 min Ar ion etching process was performed using an Ar flow rate of 20 SCCM, and RF power set at 30 W (nanoETCH, Moorfield Nanotechnology). After the cleaning step, the 150 nm thick and varied-width Al stripes (square contact/active area to GCMO) were deposited using EBPVD and lift-off. The schematic of the device configuration can be seen in Figure 1a, and a cross-section of such device in Figure 1b.

For XPS analysis, the PLD-fabricated GCMO was patterned into 400 μm wide crosspoints, with 50 nm thick Au pads, similarly as in area-dependent devices. To improve surface cleanliness, 5 s etching with HF concentration of 0.5% was performed before depositing 30 nm thick and 400 μm wide Al stripes with EBPVD and lift-off.

After ultrasonically bonding the electrical contact Al wires (HB05, TPT), the memristive properties of the fabricated devices were characterized with a Python-based program utilizing a Keithley 2614b source meter. The main electrical measurement for the characterization was repeated pulsed *IV* sweeps from 0 V → *V*_pos,max_ → 0 V → *V*_neg,max_ → 0 V, with notion *V*_pos,max_ corresponding to the situation where the Al electrode is biased more positively compared to GCMO. The sweeps included read/probe pulses between the operating voltage pulses, one suggested characterization measurement for basic memristive behavior.^32^ The chosen read pulses were −0.4 V, a low enough voltage to not induce unwanted switching in the devices.

XPS depth profiling was conducted with a Thermo Scientific Nexsa surface analysis system equipped with an Al(Kα) X-ray source and a MAGCIS dual-mode ion and cluster source. Sputtering was done iteratively in 90 s intervals with Ar^+^ ions at 300 eV, with a raster size of (0.4 × 0.8) mm^2^. After each sputtering round, XPS spectra were recorded after a 10-s delay using an X-ray spot size of 150 μm in the middle of the sputtered area. Survey spectra were recorded with 200 eV pass energy and core level spectra with 50 eV pass energy. A flood gun was used for charge compensation throughout the measurement. Al 2p and O 1s core level chemical states were analyzed and depth profiles were created with CasaXPS version 2.3.25PR1.0.^33^ Core level spectra were fitted with Gaussian–Lorentzian product type pseudo-Voigt lineshapes, defined in CasaXPS as GL(*m*), where *m* is a mixing parameter which controls the Lorentzian contribution.

## 3 Results

### 3.1 Memristive Properties

**Figure 2:**
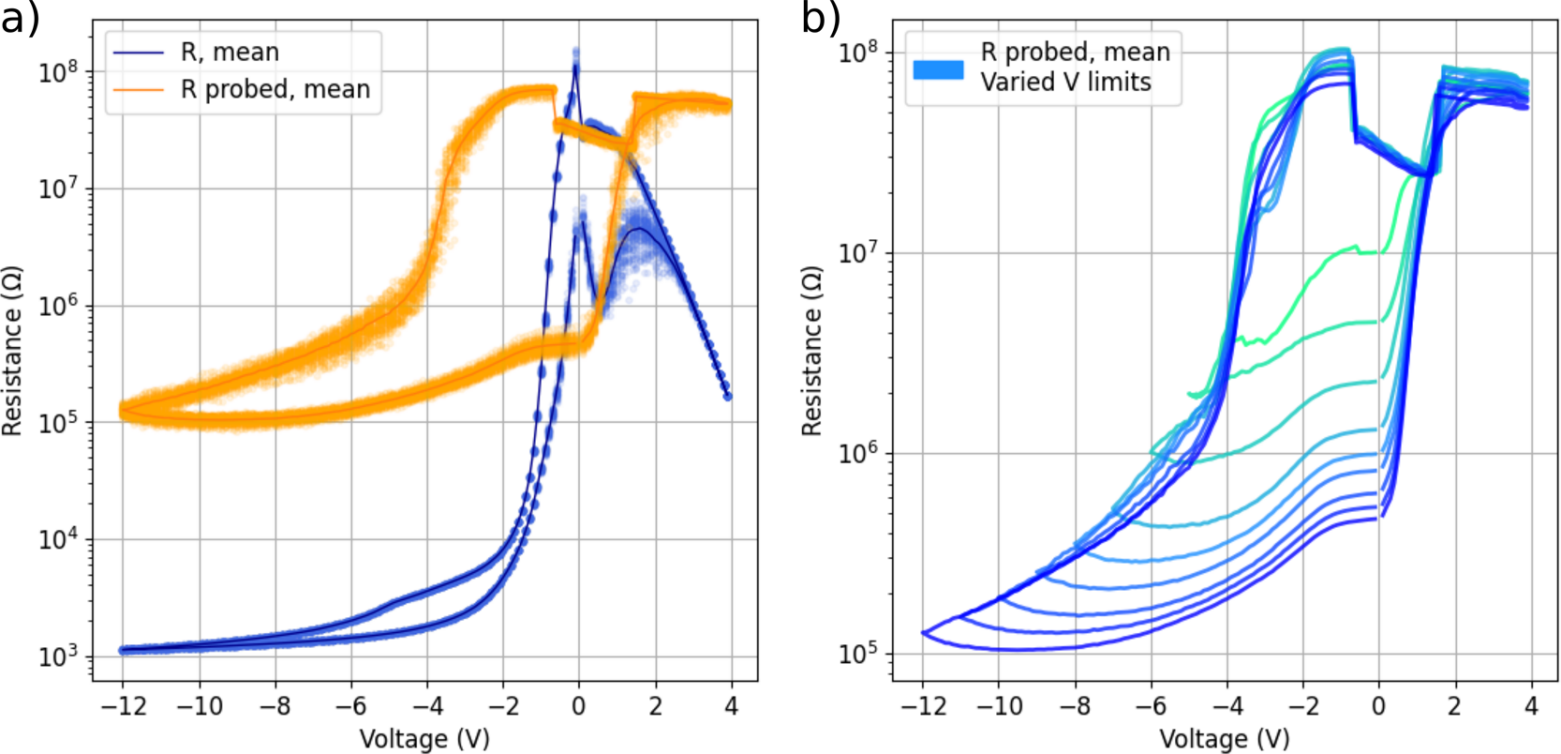
For the largest measured device (300 × 300) μm^2^: (a) an RV curve (blue) obtained from repeated 50 sweep IV measurements with a probe (−0.4 V, orange) and calculated mean (solid line) and (b) mean probe (−0.4 V) data with varied negative (SET) voltages with the mean values calculated from 50 repeated sweeps. A measurement range shift in the measurement hardware causes the indent in the data close to zero voltage.

Two devices from each varied device area were measured and displayed similar results, so only one set of various sizes is presented. Basic measurements were repeated pulsed *IV* sweeps, from which the *RV* curves with the included read/probe pulses (−0.4 V) were obtained. The mean resistance values were calculated from the 50 measured cycles. One example of such measurement result (*RV* curve) is in Figure 2a, with pulse width 20 ms, and with *V*_neg,min_ and *V*_pos,max_ voltages −12 and 4 V, respectively. It is important to acknowledge that the distinctive form of the curve near the zero-voltage region is attributable to the measurement hardware’s range shift, rather than being an inherent trait of the memristors. The *RV* curve exhibits stable, nonlinear, asymmetrical bipolar resistive switching. The devices demonstrate slight self-rectification due to the inherent nature of the rectifying Al/GCMO interface, arising from the mismatch in work functions between the aluminum electrode and the GCMO layer.

From the shapes of the *RV* curves (Figure 2a, blue curve), it is also possible to deduce the primary conduction mechanism at the Al/GCMO interface, as was done in previous works.^22^ During the set process (HRS to LRS), conduction follows the Poole-Frenkel mechanism, whereas in the reset process (LRS to HRS), HRS conduction exhibits Schottky-like behavior. The Poole-Frenkel conduction mechanism involves field-assisted electron emission from traps, which is consistent with the observed transition from HRS to LRS. Conversely, the Schottky-like behavior during the reset process suggests that conduction is dominated by thermionic emission over a barrier, aligning with the return to HRS. The probed *RV* curve (Figure 2a, orange curve) presents us with the information on how the resistive state of the device changed in response to the applied voltage pulse preceding the read operation. From this curve, we can observe the reachable resistance states and the corresponding operating voltages.

Additionally, the multistate capabilities of GCMO-based memristors were explored for potential application as synaptic weights in neural networks. By adjusting the applied voltages, particularly the negative SET voltage amplitude in the presented example, multiple distinct LRS states can be accessed, as shown in Figure 2b, where we observe one stable HRS and nine different LRS states in the mean probed data from 50 times repeated pulsed *IV* sweeps (plotted in *RV*). Unlike in filamentary systems, no compliance current is needed.^3^ This can be said to be an analog control multistate RS, viable for synaptic weight storage. The maximum HRS/LRS ratio (or dynamic range) with the investigated voltage limits and chosen read voltage was 2 orders of magnitude, achieved with the largest voltages corresponding with the strongest applied electric field. When the Al electrode is biased at a higher negative voltage compared to GCMO, the resultant stronger electric field increases the mobility of oxygen ions, directing them from the Al electrode into the GCMO layer, setting a new lower resistance state. This reduces the oxidation at the Al/GCMO interface, thereby decreasing the interface resistance. Similarly, when Al is biased positively, the interface oxidation increases and resets the device. For optimal performance in synaptic networks, both the dynamic range, which should exceed 1 order of magnitude, and the multistate capability, i.e., the number of distinguishable states, must be sufficiently high.^1^

**Figure 3:**
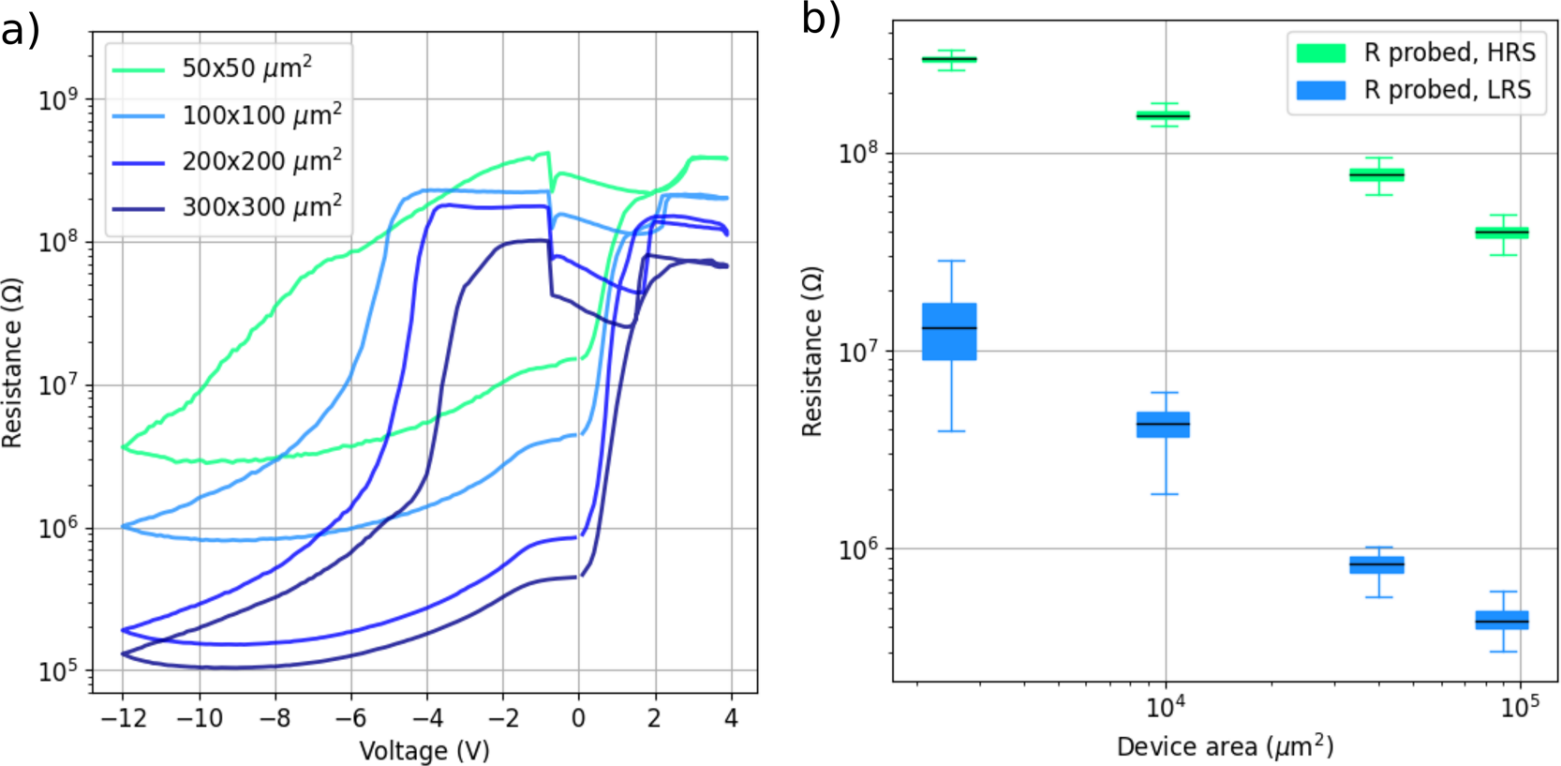
(a) Mean probe (−0.4 V) data with varied device areas, 4 devices total, mean values calculated from 100 repeated sweeps. A measurement range shift in the measurement hardware causes the indent in the data close to zero voltage. (b) Box plot for HRS and LRS states with varied device areas, 4 devices total. Values extracted from repeated 100 sweeps probed data at −0.4 V.

To investigate how the device resistances (both high- and low-resistance states, HRS and LRS respectively) scale as a function of the active area, the mean probed (−0.4 V) resistances from 100 times repeated *IV* sweeps were depicted in Figure 3a, and a box plot of resistance values extracted from the probed data in Figure 3b. The measured average resistances for HRS ranged from 10^7^ to 10^8^ Ω, and for LRS they ranged from 10^5^ to 10^7^ Ω. As the device’s active area decreases, the interface resistance increases in both LRS and HRS. The LRS resistances follow a clear linear behavior, with the slope around −1 Ω/μm^2^. In HRS, the area-dependence also follows the −1 Ω/μm^2^ slope for the two largest devices, but deviates from that in the smallest two due to the high resistances causing issues in our measurement system. An alternate explanation for the HRS resistance slope differing from the expected −1 is the increased edge effects of the top or active electrode. When the device is in HRS, the current flow may be concentrated at the edges of the top electrode due to the enhanced electric field in these regions, pronouncing the edge effect as the electrode area decreases. In contrast, when the device is in LRS, the current flow could be more uniformly distributed across the entire electrode area, mitigating the edge effect and resulting in a slope closer to the expected.^6^ These proposed edge effects could also influence the measured responses of devices with different sizes, particularly altering the shape of the curves in Figure 3a, primarily on the set side.

This inverse proportionality is evidence for interface type RS but can also indicate bulk type RS.^2^ Similar area-dependent behavior has been reported in PCMO-based devices,^34,35^ where relatively large device areas were explored, similar to those in our work. Additionally, the resistance scaling effect is also known to continue to nanoscale dimensions.^11^ It is proposed, that device memory performance, such as retention, dynamic range, and switching energy, strongly improve when device size is decreased from a large area (μm-scale) to nanoscale.^11,12^ The expected behavior in the presence of conductive filaments is constant LRS resistance values over different device active areas, so determining the area-dependence is the first step for ruling out filamentary RS.^36,37^ To rule out the effect of the other interface, it is important to select a passive electrode material. The Ohmic nature of the Au/GCMO interface is well-documented in previous research on GCMO-based memristors.^22^ Similar devices across the full Ca-doping range were analyzed, demonstrating that the Au/GCMO/Au device forms an Ohmic contact due to the similar work functions of the two materials.

Typical for interfacial switching is the relaxation of LRS state over time.^3^ A sudden, slight relaxation has also been observed with previous Al/GCMO memristors, preceding retention times measured up to 8 h.^24,25^ This behavior supports the hypothesis that the RS in GCMO originates from ion migration, notably oxygen ions/vacancies moving near the interfacial region. As the relaxation of LRS state in synaptic applications is not sought after, some further interface engineering could be implemented into the process, as has successfully been demonstrated with PCMO.^11^ While the relaxation behavior highlights challenges associated with interfacial dynamics rooted in ionic movement, these same dynamics contribute to GCMO’s versatility. For instance, GCMO-based memristors have demonstrated spike-timing-dependent plasticity (STDP), a key feature for spiking neural networks (SNNs), further endorsing their suitability for neuromorphic applications.^23^

Previous studies with GCMO as the active electrode in a memristor have demonstrated robust retention characteristics, with states remaining well distinguishable for up to 8 h.^22,24^ In our study, while no official retention measurement was conducted before the XPS depth profiling, the stability of the states was assessed by a quick read pulse preceding the following XPS measurement. The states had been set the day before, and we observed that the retention was sufficient for the XPS measurements.

### 3.2 XPS Depth Profiling

For in-depth analysis of XPS depth profiles, one representative HRS device and one LRS device were chosen. We opted to omit the pristine, nonelectrically characterized devices from the XPS depth profiling comparisons, as the devices are initially after fabrication in HRS and start exhibiting RS with no added forming step. Chemical state analysis was done by peak fitting to Al 2p and O 1s core level spectra, as those are the main peaks displaying the differences between the examined HRS and LRS devices. First, a Shirley background was subtracted from each spectrum.

**Figure 4:**
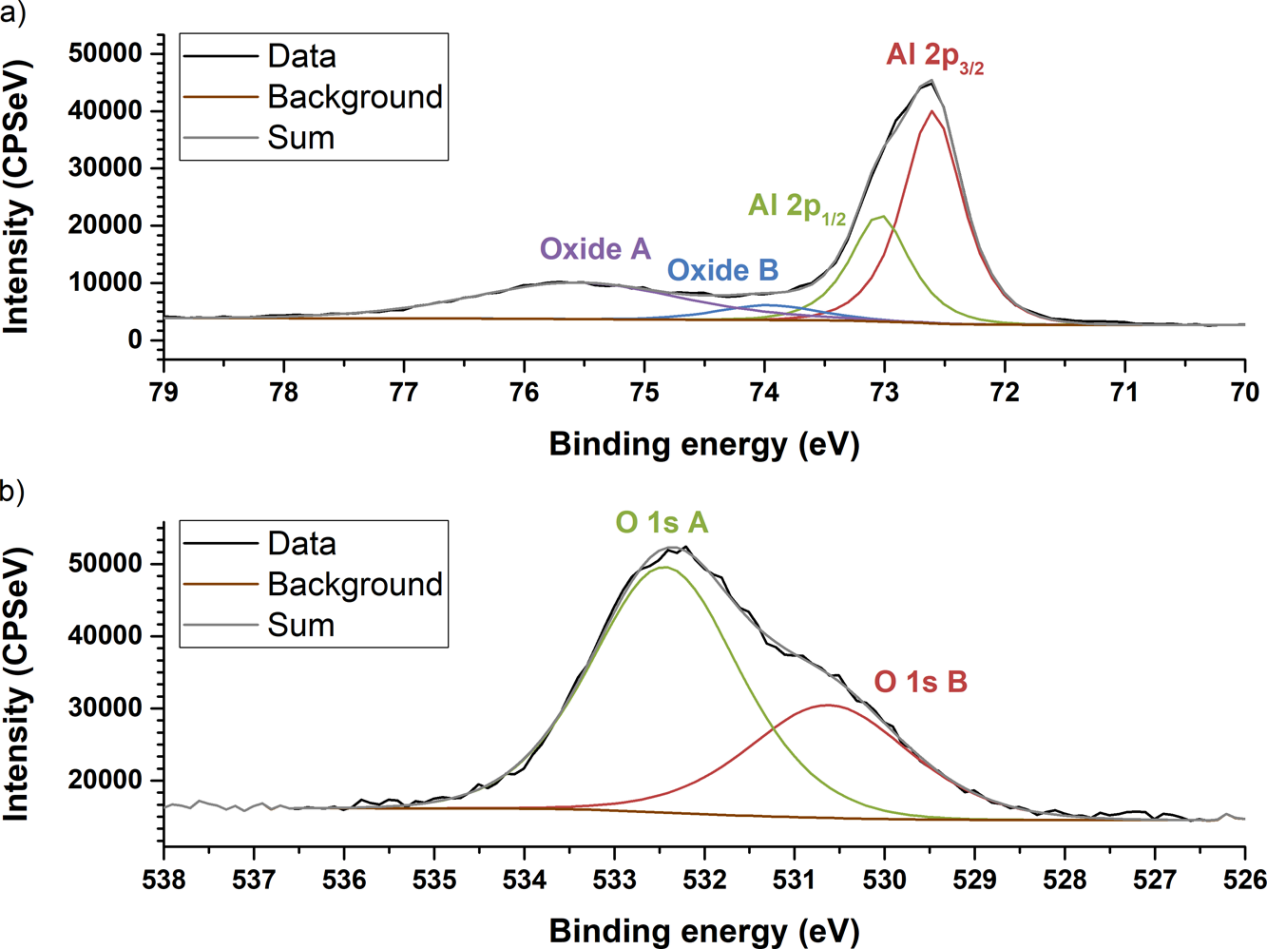
Examples of peak fitting to (a) Al 2p and (b) O 1s core-level spectra. Both spectra are recorded after 1260 s of sputtering.

The Al 2p spectra were fitted with four components, corresponding to Al 2p_3/2_, Al 2p_1/2_, and two Al oxide peaks. A GL(80) line shape was used for the metallic Al components and a GL(50) line shape for the oxide components.^38^ Al 2p spin–orbit splitting was constrained to 0.44 eV,^39^ the Al 2p_3/2_:Al 2p_1/2_ ratio was constrained to 2:1, and the metallic components’ full width half maxima (fwhm) were set equal to each other. Figure 4a presents an example fit to an Al 2p core level spectrum after 1260 s of sputtering. The Al 2p_3/2_ and Al 2p_1/2_ components arise from metallic aluminum. The metallic components were removed from the spectra after the Al/GCMO interface was reached to prevent false fittings to noise, at 2520, and 2160 s of sputtering time in HRS and LRS, respectively. The component labeled Oxide A at higher binding energy arises from Al_2_O_3_ and component Oxide B at lower binding energy arises from other intermediate aluminum oxides.^40^

The O 1s spectra were fitted with two components. A GL(50) line shape was used for both components.^38^ The higher binding energy component is annotated as A, and the lower binding energy component is annotated as B. The component A at higher binding energy corresponds to Al–O bonds in spectra recorded near the surface, but deeper at the Al/GCMO interface, it begins to include contributions from oxygen lattice defects.^41−43^ Because Al–O contributions and oxygen lattice defect contributions are very close to each other in binding energy, it is not possible to separate their contributions at the interface. Component B at lower binding energy corresponds to GCMO lattice oxygen.^41−43^ Component B was added to the fit at the level where Ca 2p signal became visible, indicating that the sputtering had progressed to the Al/GCMO interface. Figure 4b presents an example of fitting for an O 1s core level spectrum.

**Figure 5:**
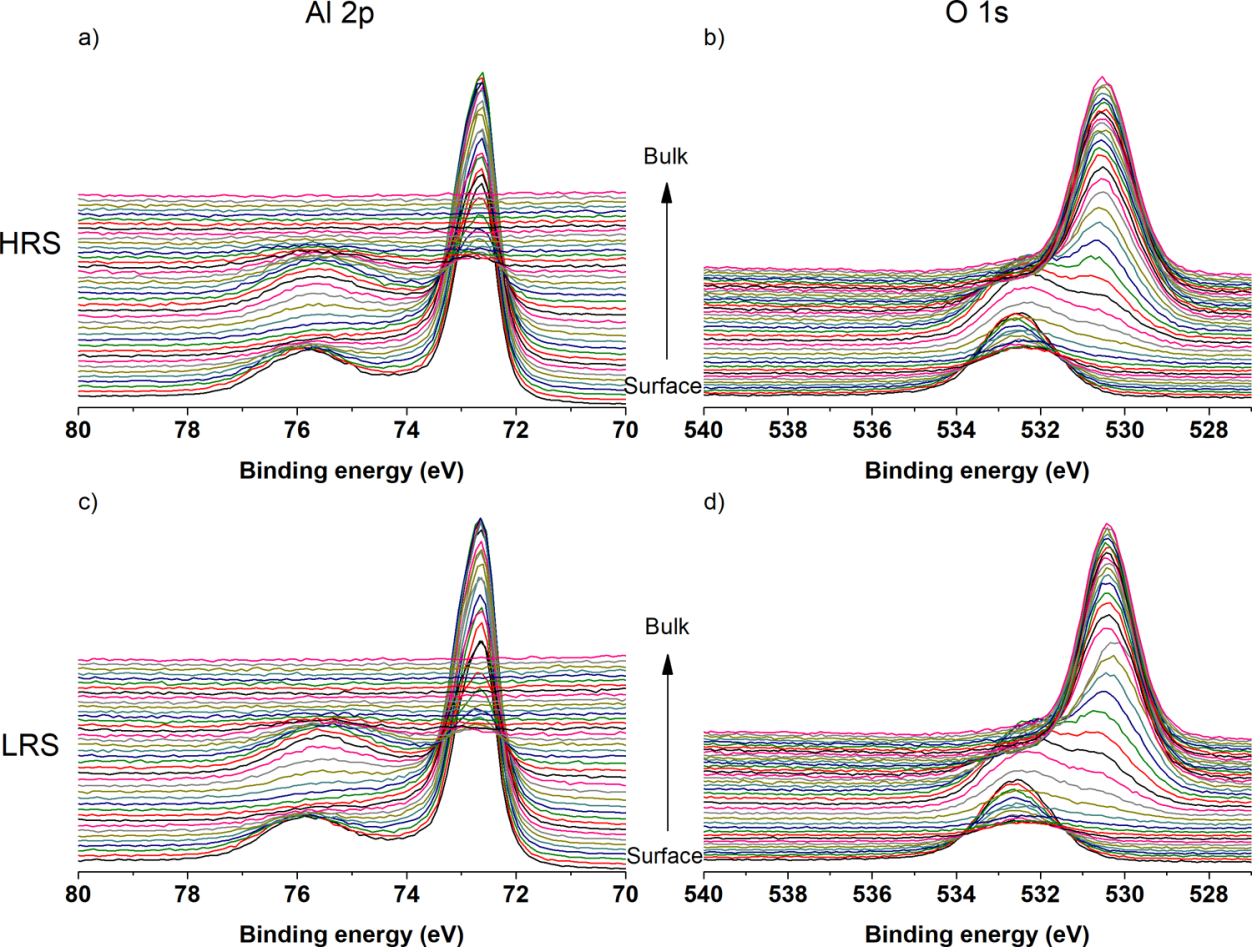
Montages of core-level spectra. (a) Al 2p core-level spectra from the HRS device, (b) O 1s core-level spectra from the HRS device, (c) Al 2p core-level spectra from the LRS device, and (d) O 1s core-level spectra from the LRS device.

After peak fitting, charge correction was performed for spectra in each depth profile level. Adventitious carbon could not be used as charge reference, because the sample surface contained very little carbon. Moreover, the carbon concentration inside the Al/GCMO stack is not detectable in these measurements. Despite using the flood gun throughout the profiling, the interactions of the sample with the sputter ion beam may cause charging,^44^ and each depth profile level must be calibrated. Therefore, the energy calibration at each depth profile level was done with respect to the metallic Al 2p_3/2_ peak, whose position was fixed at 72.6 eV.^39^ However, the Al 2p_3/2_ peak disappears as expected when the GCMO bulk is reached, so there the calibration was performed with respect to the O 1s component B, which arises from GCMO lattice oxygen and can be assumed to appear at a constant binding energy throughout the stack. The O 1s B energy was related to the Al 2p_3/2_ by calculating the average of their energy differences from the spectra in the interface region where both components appear, between 1711 and 1981 s, and 1531 and 1890 s of sputtering time in HRS and LRS, respectively. The calibration was performed relative to Al 2p_3/2_ until 1980, and 1890 s in HRS and LRS, respectively. In deeper levels, the calibration was performed relative to O 1s B. The O 1s B energy was fixed at 530.5 eV. Montages of Al 2p and O 1s core level spectra are presented in Figure 5.

**Figure 6:**
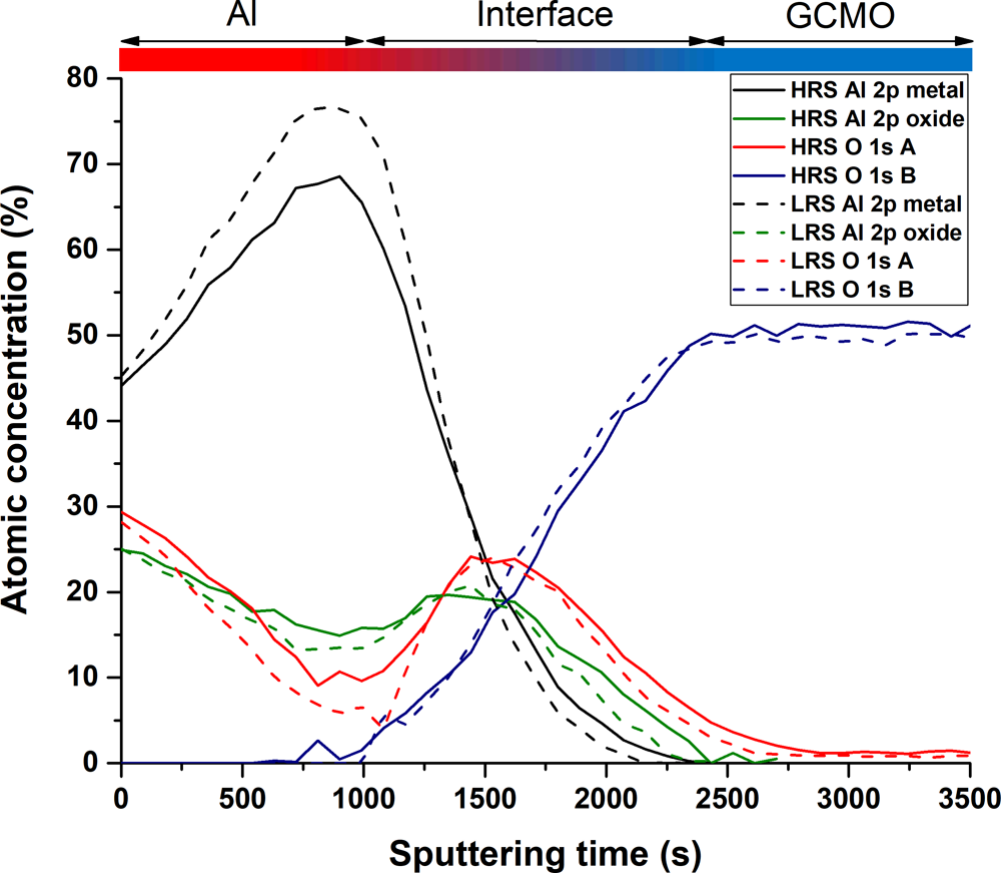
Depth profile of Al 2p and O 1s chemical states for a representative HRS and LRS device. Atomic concentration is calculated as a percentage of total; however, elements other than Al and O are omitted for clarity.

Depth profiles of the HRS and LRS devices’ Al 2p and O 1s chemical states were constructed based on these fits. We focus on the differences between the metallic and oxide Al 2p components, so the metallic Al 2p_3/2_ and Al 2p_1/2_ components as well as the oxide A and oxide B components in the Al 2p region were summed together for the analysis. Depth profiles from the HRS and LRS devices are presented in Figure 6.

The depth profiles can be divided into three distinct regions: the aluminum pad (Figure 6 leftmost side), the GCMO (Figure 6 rightmost side), and an interface region in between. The interface region is defined here as the region between the sputtering level where the GCMO lattice oxygen (O 1s component B, blue solid/dashed lines) first appears, and the level where the GCMO lattice oxygen first reaches its bulk concentration. Therefore, the interface region covers sputtering times of around 600 to 2300 s in HRS and around 900 to 2300 s in LRS. The interface region is wide both due to element interdiffusion and XPS information depth.

The metallic aluminum component (Al 2p metal, black solid/dashed lines) is dominant in the top layers of the Al pad and first increases with sputtering depth, after which the component decreases and disappears as expected from the device geometry. The peak of the Al 2p metal concentration occurs at around 1000 s of sputtering in both HRS and LRS, but the key difference lies in the oxidation behavior: In HRS, the Al 2p oxide fraction (green solid line) is higher than in LRS (green dashed line), indicating stronger oxidation of aluminum in the high-resistance state. This increased oxidation coincides with the O 1s A component (red solid line), which is associated with both Al–O contributions and oxygen defect contributions in the GMCO lattice. The interface region in HRS appears broader than in LRS, evidenced by the more gradual decay of metallic Al 2p and the slower rise of O 1s components, suggesting enhanced interdiffusion between Al and GCMO. In contrast, LRS exhibits a sharper transition between the Al pad and GCMO, with lower oxidation and a more defined interface, indicating less oxygen migration.

The depth profiles show that aluminum is oxidized more in the HRS sample than in the LRS one in the middle of the aluminum pad around the 1000-s mark. This is evident from the atomic concentrations of the O 1s A component and Al oxide components in HRS compared to LRS. The increased oxidation of interfacial aluminum in HRS is consistent with the hypothesis that oxygen migrates from GCMO toward the aluminum electrode during the resistive switching process. As the top surface oxidation of the Al pad is similar in both HRS and LRS devices, the differences observed close to the interface arise from their inherent differences formed in the HRS-to-LRS transition. Because oxygen is highly mobile in perovskite manganite structures,^28,45^ it can diffuse toward the Al pad from a depth that is below the last measured XPS depth profiling level. This suggests that even though a direct comparison of GCMO lattice oxygen concentration between HRS and LRS at a specific sputtering depth may not reveal a large difference, the cumulative migration of oxygen over time is responsible for the increased oxidation observed at the Al/GCMO interface in HRS. A broadened Al/GCMO interface is also observed in the XPS profiles for HRS compared to LRS, which supports an increased diffusion of elements over the interface for the HRS sample. The interface broadening could also indicate a progressive oxidation process in HRS, leading to a more extended transition region between metallic aluminum and GCMO, thus resulting in an increased insulating barrier for current.

## 4 Discussion

The electrical characterization and XPS depth profiling together provide a thorough understanding of the RS mechanism in GCMO-based memristors. The observed area-dependent behavior of HRS and LRS resistances strongly supports an interface-type RS mechanism, where resistance scales with the device area. This scaling is inversely proportional, with linear fitting yielding a slope of approximately −1. Additionally, the multistate capability of the devices suggests a gradual evolution in the formation of aluminum oxide at the interface, which is influenced by the applied electric field.

The insights from the memristive measurements are further validated by the XPS depth profiles, which highlight distinct differences in the chemical states between HRS and LRS. The increased oxide content in the Al pad and increased diffusion of oxidized species across the Al/GCMO interface in HRS suggest that oxygen ion migration at the Al/GCMO interface is critical in modulating the resistive states. The depth profiles allow a detailed examination of chemical states in three distinct regions: the aluminum pad, the GCMO layer, and the interfacial region in between.

The interfacial processes driving the RS behavior offer several advantages, particularly the ability to achieve multistate RS through voltage modulation. This underscores GCMO’s potential as an ideal material for synaptic weight storage, enabling analog control in neuromorphic computing systems. Together, these findings emphasize the suitability of GCMO-based memristors, driven by interfacial dynamics, for high-density crossbar arrays, which are vital for neuromorphic architectures.

## 5 Conclusions

We have demonstrated that GCMO-based memristors exhibit area-dependent resistive switching behavior, indicative of interface-driven mechanisms suitable for high-density crossbar arrays in neuromorphic systems. Results from XPS depth profiling support the role of oxygen diffusion and interfacial AlO_*x*_ layer in modulating resistive states, with a distinction in overall oxide content and the width of the interfacial region in the Al/GCMO memristor devices. Furthermore, the ability to achieve multistate RS through voltage modulation illustrates GCMO’s adaptability for analog synaptic weight adjustments in artificial neural networks. Future work could focus on interface engineering to mitigate LRS relaxation to improve state stability and enhance the dynamic range of resistive states. Additionally, exploring nanoscale device fabrication could unlock improved performance in terms of retention and endurance, addressing scalability challenges for next-generation computing architectures. The demonstrated multistate RS capability also opens pathways for implementing GCMO-based memristors in complex neuromorphic systems, where analog synaptic weight modulation is essential for learning algorithms and real-time processing. These insights position GCMO as a compelling material for next-generation neuromorphic computing architectures.
